# Phylogenetic Analysis of Algal Symbionts Associated with Four North American Amphibian Egg Masses

**DOI:** 10.1371/journal.pone.0108915

**Published:** 2014-11-13

**Authors:** Eunsoo Kim, Yuan Lin, Ryan Kerney, Lili Blumenberg, Cory Bishop

**Affiliations:** 1 Division of Invertebrate Zoology and Sackler Institute for Comparative Genomics, American Museum of Natural History, New York, New York, United States of America; 2 Department of Biology, St. Francis-Xavier University, Antigonish, Nova Scotia, Canada; 3 Department of Biology, Gettysburg College, Gettysburg, Pennsylvania, United States of America; University of Cambridge, United Kingdom

## Abstract

Egg masses of the yellow-spotted salamander *Ambystoma maculatum* form an association with the green alga “*Oophila amblystomatis*” (Lambert ex Wille), which, in addition to growing within individual egg capsules, has recently been reported to invade embryonic tissues and cells. The binomial *O. amblystomatis* refers to the algae that occur in *A. maculatum* egg capsules, but it is unknown whether this population of symbionts constitutes one or several different algal taxa. Moreover, it is unknown whether egg masses across the geographic range of *A. maculatum*, or other amphibians, associate with one or multiple algal taxa. To address these questions, we conducted a phylogeographic study of algae sampled from egg capsules of *A. maculatum*, its allopatric congener *A. gracile*, and two frogs: *Lithobates sylvatica* and *L. aurora*. All of these North American amphibians form associations with algae in their egg capsules. We sampled algae from egg capsules of these four amphibians from localities across North America, established representative algal cultures, and amplified and sequenced a region of 18S rDNA for phylogenetic analysis. Our combined analysis shows that symbiotic algae found in egg masses of four North American amphibians are closely related to each other, and form a well-supported clade that also contains three strains of free-living chlamydomonads. We designate this group as the ‘*Oophila*’ clade, within which the symbiotic algae are further divided into four distinct subclades. Phylogenies of the host amphibians and their algal symbionts are only partially congruent, suggesting that host-switching and co-speciation both play roles in their associations. We also established conditions for isolating and rearing algal symbionts from amphibian egg capsules, which should facilitate further study of these egg mass specialist algae.

## Introduction

“*Oophila amblystomatis*” is a binomial corresponding to the chlamydomonad green alga that lives in association with several species of North American amphibian embryos [1]. These amphibians include the ambystomatid salamanders *Ambystoma maculatum* (spotted salamander) and *A. gracile* (Northwestern salamander), and ranid frogs *Lithobates sylvatica* (wood frog) and *L. aurora* (red-legged frog) [2]. This alga was provisionally named by Lambert, who collected and preserved samples of the algal cells from *A. maculatum* embryos in 1905, north of Boston [3]. The use of the informal designation “*Oophila amblystomatis*” has remained in the scientific literature, with the occasional spelling of “*Oophilia*” [4]–[10], despite the lack of a formal taxonomic description. Additionally, while many researchers accept the chlamydomonad designation for *Oophila*, it remains unknown whether the algae consist of a mono- para- or polyphyletic group within and among these different amphibian hosts [2].

Most research on *O. amblystomatis* has focused on just one of these hosts, the spotted salamander *A. maculatum*. The *A. maculatum*-algal association was documented over 125 years ago [11], and the majority of subsequent research on this association focused on the reciprocal benefits to the algae and host [4]–[6], [12]–[16]. The embryo benefits from (i) an increase in the partial pressure of oxygen in its egg capsules provided by the algae during daylight hours [6], (ii) potential removal of nitrogenous waste ([12] although see [15]), and (iii) potential transfer of photosynthate from algae to embryos [16]. Other potential benefits to the embryos may include reduced production of carbonic acid (H_2_CO_3_), as a result of reduced CO_2_ inside the egg capsule, or the direct or indirect exclusion of microbial pathogens by the algae. Benefits for the algae may include the provision of nitrogenous wastes in the form of ammonia by the hosts [12], the increased CO_2_ within the egg capsule, as well as a protective environment for the algae to flourish.

Past researchers considered this association to be ectosymbiotic (i.e. inside the egg capsule, but outside the body), but Kerney et al. [17] recently showed that algal cells invade host embryonic tissues and cells. This unique example of algal endosymbiosis in a vertebrate host cells raises many immediate research questions. Is *O. amblystomatis* the sole algal symbiont associated with *A. maculatum*? Are there signs of co-evolution between symbiont and host? Finally, are the symbionts of other North American amphibians closely related to those found associating with *A. maculatum*? Answers to these questions will advance our understanding of the nature of this intriguing association.

This study employs a phylogeographic approach to better understand the identity and relationships among algae that form symbiotic associations with North American amphibians. To this end, we chose 18S rDNA as a marker as this gene has been broadly sampled across eukaryotic algae (e.g. the SILVA rRNA database [18]) and hence is suitable for placing taxa of interest to major phylogenetic groups (e.g. [19]–[20]). We inferred an 18S rDNA phylogeny from environmental samples of algae collected from *Ambystoma maculatum, A. gracile, Lithobates sylvatica*, and *L. aurora* egg capsules. We also included in our analysis three “*Chlamydomonas gloeophila*,” (Skuja) strains, which were obtained from *A. maculatum* egg masses in the Northeastern USA in the early 1950' or as free-living cells from a freshwater body in England. We found algae, which associate with embryos of these four amphibian taxa, form a clade together with three cultured strains of chlamydomonad taxa, but not *C. gloeophila*. We suggest that the genus *Oophila* is accurately assigned to this discrete algal lineage. Within this group, amphibian-associated algae fall into four possible subclades that do not strictly correspond to their host species.

## Materials and Methods

### Collection and preparation of samples

**Figure 1:**
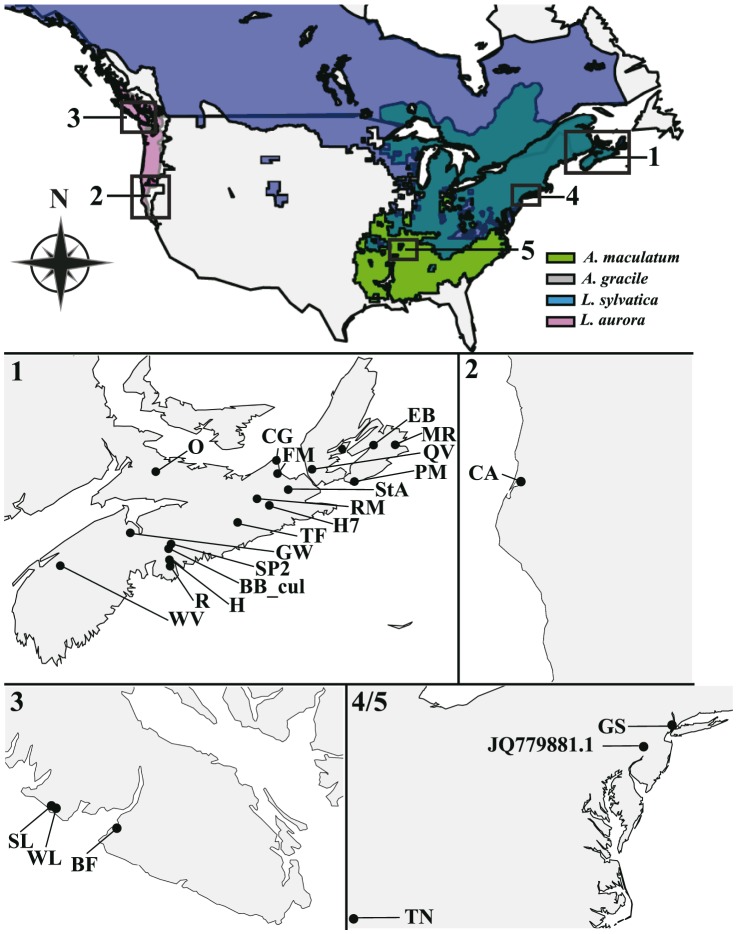
Map of the geographic range and collection sites for egg masses of four amphibian hosts. Species range maps are plotted on a map of North America (see the Materials and Methods). The dark green color represents a range overlap between *L. sylvatica* and *A. maculatum*, and the pink color represents a range overlap between *L. aurora* and *A. gracile*. Numbered locations correspond to higher detail panels below. The maps of collection sites for algae corresponding to egg masses from *A. maculatum* and L. sylvatica in Nova Scotia, Canada (1), *A. gracile* in California, USA (2), *L. aurora* and *A. gracile* in Vancouver Island, British Columbia, Canada, and *A. maculatum* in New Jersey and Tennessee of USA (4/5).

Egg clutches of amphibians were collected from vernal ponds or other types of temporary or permanent freshwater bodies between 2009 and 2013. Collection sites included multiple locations from New Jersey (USA), Tennessee (USA), California (USA), British Columbia (Canada), and Nova Scotia (Canada) (Figure 1, Table 1). None of the amphibian species from whose egg masses algae were collected are endangered or protected. Collections were approved as part of Animal Care Protocols to RK (IACUC#2013F17; Gettysburg College Animal Care Committee) and CB (CCAC#12-007-N; St. Francis-Xavier Animal Care Committee). Most samples were from locations for which no specific permission was required. Samples from the Greenbrook Sanctuary (private land) were collected with permission (Sandra Bonardi, Director) and samples from the University of the South (private land) were collected by Professor David G. Haskell. Nova Scotia and British Columbia Ministry of Natural Resources granted permission for collections. For NS collections, a letter from the relevant authorities, but no permit number is issued. For BC collections, permits #NA11-68662 was issued to RK for *A. gracile*; #NA12-76509 to CB for *L. aurora*). Sampling locations, including GPS co-ordinates are listed in Table 1. Typically, clutches were collected when the algal bloom inside each egg was visible, which occurs after Harrison stage 17 [17], [21]. Algal cells were collected by piercing an egg and withdrawing the capsular fluid using an insulin syringe, or by dissecting out the capsular part of the egg with fine forceps. These collection methods constitute environmental samples, since no selection of cells was performed prior to DNA extraction. *C. gloeophila* strains were obtained from the Experimental Phycology and Culture Collection of Algae at the University of Göttingen (strains SAG 12–4, 12–5) and the Culture Collection of Algae and Protozoa, maintained by the Scottish Association of Marine Science (strain 11/127).

**Table 1:** Collection details of egg masses from which algae were sampled.

Location	Code	Coordinates (dec. degrees)		Date	Sample type	Host	GenBank Acc.
***Nova Scotia***		lon	lat				
St. Andrews	StA1	−61.750	45.499	01-04-11	ES*	*A. maculatum*	KJ711228, KJ711229, KJ711248
St. Andrews	StA2	−61.750	45.499	05-05-12	ES	*A. maculatum*	KJ711230, KJ711231, KJ711232
East Bay	EB	−60.362	46.013	25-04-11	ES	*A. maculatum*	KJ711198, KJ711238
Graywood (Hwy 8)	GW	−65.426	44.622	14-05-11	ES	*A. maculatum*	KJ711202
Wolfville	WV	−64.297	45.001	13-05-11	ES	*A. maculatum*	KJ711236, KJ711237
Hwy#7	H7	−62.055	45.318	21-04-12	ES	*A. maculatum*	KJ711242, KJ711205, KJ711206
Fairmont	FM	−61.922	45.684	26-04-12	ES	*A. maculatum*	KJ711199, KJ711200, KJ711201
Snakepit 2	SP2	−63.639	44.872	28-04-12	ES	*A. maculatum*	KJ711226, KJ711227, KJ711246, KJ711247
Trafalgar	TF	−62.566	45.121	29-04-12	ES	*A. maculatum*	KJ711233, KJ711234, KJ711235
Queensville	QV	−61.366	45.734	29-04-12	ES	*A. maculatum*	KJ711215, KJ711216, KJ711217, KJ711218, KJ711219
Rocky Mountain	RM	−62.253	45.394	02-05-12	ES	*A. maculatum*	KJ711220, KJ711221, KJ711222
Mira River	MR	−60.015	46.017	05-05-12	ES	*A. maculatum*	KJ711207, KJ711208, KJ711209
Oxford	O	−63.886	45.707	11-05-12	ES	*A. maculatum*	KJ711210, KJ711211, KJ711243
Point Michaud	PM	−60.668	45.593	22-05-12	ES	*A. maculatum*	KJ711212, KJ711213, KJ711214
Cape George	CG	−61.941	45.836	28-05-12	ES	*A. maculatum*	KJ711196, KJ711197
Halifax	Hb_cul	−63.654	44.617	2011	CI**	*A. maculatum*	KJ711137
Halifax	H	−63.666	44.691	2009	ES	*A. maculatum*	KJ711203, KJ711204, KJ711239, KJ711240, KJ711241
Beaver Bank	BB_cul	−63.680	44.818	2011	CI	*A. maculatum*	KJ711131, KJ711132, KJ711134, KJ711135, KJ711136, KJ711138
Halifax	R	−63.654	44.617	2011	ES	*L. sylvatica*	KJ711139, KJ711140, KJ711141, KJ711142, KJ711143, KJ711144, KJ711145, KJ711146, KJ711147
***British Columbia***							
Bamfield	BF	−125.016	48.871	05-05-11	ES	*A. gracile*	KJ711133, KJ711162, KJ711163, KJ711164, KJ711165, KJ711166, KJ711167, KJ711168, KJ711169, KJ711170, KJ711171, KJ711172, KJ711173
Wood Lake	WL	−125.581	48.991	24-03-12	ES	*L. aurora*	KJ711223
Swan Lake	SL	−125.595	49.001	21-03-12	ES	*L. aurora*	KJ711224, KJ711225, KJ711244, KJ711245
***New Jersey, USA***							
Greenbrook Sanctuary	GSa	−73.924	40.914	17-03-12	ES	*A. maculatum*	KJ711181, KJ711182, KJ711183, KJ711184, KJ711186, KJ711187, KJ711188, KJ711189, KJ711190, KJ711193
Greenbrook Sanctuary	GSb	−73.924	40.914	25-03-12	ES	*A. maculatum*	KJ711174, KJ711175, KJ711176, KJ711177, KJ711194
Greenbrook Sanctuary	GSc	−73.924	40.914	14-04-12	ES	*A. maculatum*	KJ711178, KJ711179, KJ711180, KJ711185
Greenbrook Sanctuary	GSa_cul	−73.924	40.914	17-03-12	CI	*A. maculatum*	KJ711195
Greenbrook Sanctuary	GSb_cul	−73.924	40.914	25-03-12	CI	*A. maculatum*	KJ711191, KJ711192
***Tennessee, USA***							
Sewanee	TN	−85.910	35.204	02-18-13	ES	*A. maculatum*	KJ711249, KJ711250, KJ711251, KJ711252, KJ711253, KJ711254, KJ711255, KJ711256
***California, USA***							
Arcata	CA	−124.082	40.867	03-07-12	ES	*A. gracile*	KJ711148, KJ711149, KJ711150, KJ711151, KJ711152, KJ711153, KJ711154, KJ711155, KJ711156, KJ711157, KJ711158, KJ711159, KJ711160, KJ711161
***Culture collections***							
*C. gloeophila*	SAG 12-4	−86.568	39.143	1953	CI	*A. maculatum*	KJ711128
*C. gloeophila*	SAG 12-5	−76.475	42.447	1953	CI	*A. maculatum*	KJ711129
*C. gloeophila*	CCAP 11/127	−2.935	54.381	1992	CI	Free-living	KJ711130

*ES  =  environmental sequence; **CI  =  cultured isolate.

**Figure 2:**
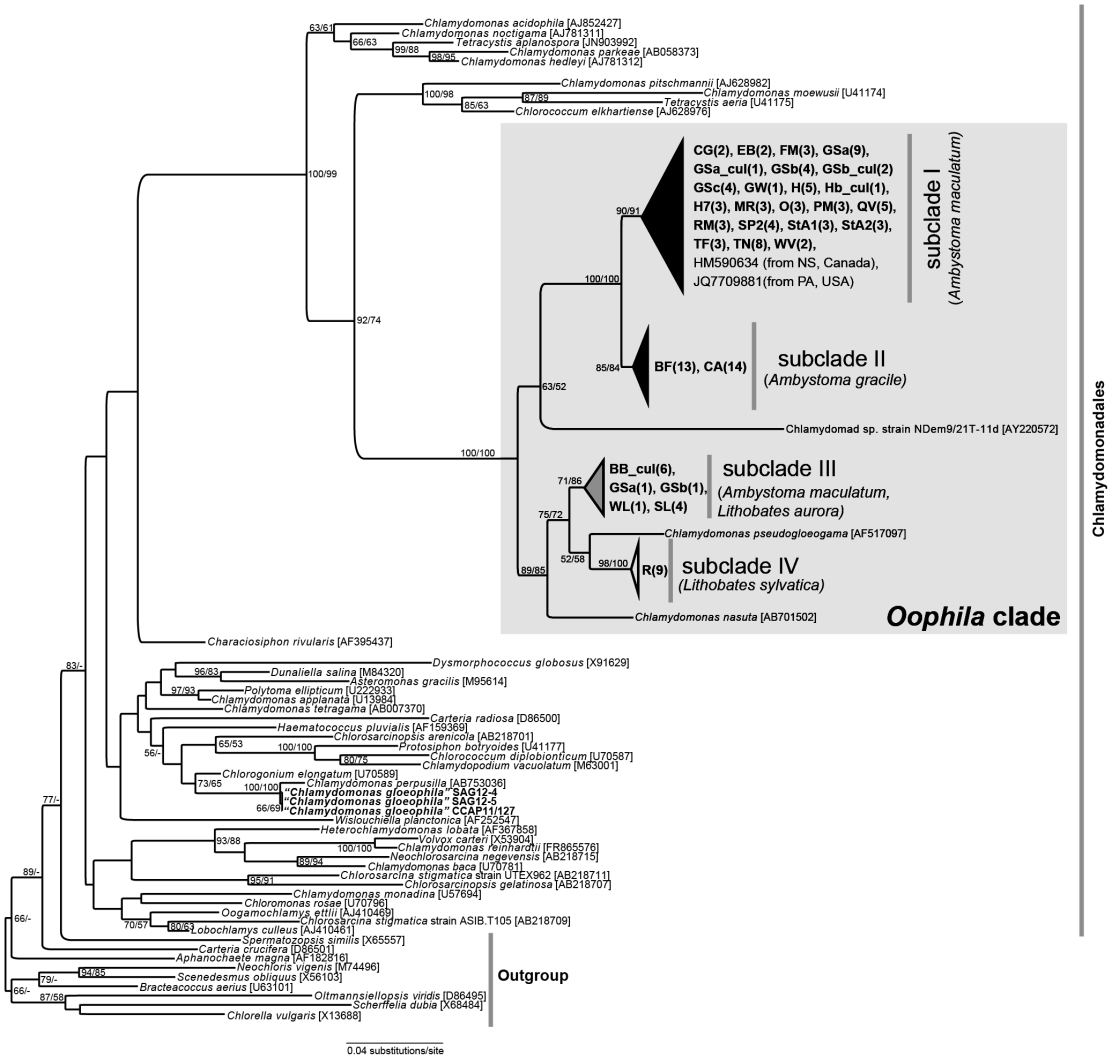
Maximum likelihood (ML) tree of algal 18S rDNA sequences from egg masses of four amphibian taxa from various North American localities. The data matrix included 1,653 characters and 180 sequences. Newly obtained sequences are bold-faced. ML and MP bootstrap values greater than 50% are shown at corresponding nodes. Subclades I−IV are collapsed into triangles for visual clarity; an un-collapsed version of the tree can be found as Figure S1. Numbers in parentheses indicates the number of sequences obtained and analyzed for the corresponding sample. See Table 1 for naming conventions and GenBank accession numbers.

Name codes correspond to branch names in Figure 2.

### Map Construction

Maps were plotted using maps (ver. 2.3−2; http://cran.r-project.org/projects/maps/), mapdata (ver. 2.2−2; http://cran.r-project.org/projects/mapdata/) and maptools (ver. 0.8.23; http://cran.r-project.org/projects/maptools/) packages implemented in R (ver. 3.0.1) [22]. Host species range maps were downloaded from the IUCN *Red List of Threatened Species* (ver. 2012.2 http://www.iucnredlist.org/) [23]–[26]. Sample collection sites were recorded using GPS or in cases where GPS co-ordinates were not recorded, were estimated using a geographic locator (http://www.findlatitudeandlongitude.com/). Collection locations on the maps were annotated in Adobe Illustrator CS5 (ver.15.1.0).

### Culturing and microscopy of algal symbionts

Algal cells associated with *A. maculatum* egg capsules were isolated using a finely drawn Pasteur pipette. In addition to single cell isolation, cultures of green algae were established by serial dilution techniques using disposable multi-well plates. These were cultured in modified AF6 medium [27]. Whereas we also used 0.5−1% agar solidified AF6 medium for culturing, the symbiotic algae grew very poorly on an agar plate (see the discussion for further details). Cultures were maintained in a plastic tube with vent screw cap and at 15−20°C with a 12-hour light cycle, under broad-spectrum growth lights. Cultures were transferred aseptically every 4−8 weeks. Algae were imaged using an Axiovert 100 microscope (Zeiss, Oberkochen, Germany) and an Olympus DP73 digital camera (Tokyo, Japan). The Olympus cellSens image capture software was used to measure the cells.

### Molecular sequencing and phylogenetic analysis

From each individual egg, the capsular algae were processed for DNA extraction using a DNeasy Blood and Tissue kit, or in some cases, a DNeasy Plant tissue kit (Qiagen, Hilden, Germany). The extracted DNA was used as a template for PCR using sets of “universal” eukaryotic 18S rDNA primers, including nu-SSU-0024-5′ (5′-CTGGTTGATCCTGCCAGTAGT-3′), nu-SSU-0033-5′ (5′- CCTGCCAGT AGTCATAYGCTT-3′), nu-SSU-1757-3′ (5′-CAGGTTCACCTACGGAAACCT-3′), and nu-SSU-1768-3′ (5′- TGA TCC TTC YGC AGG TTC ACC-3′) [28]. Amplified products were purified using a QIAquick gel extraction kit (Qiagen) and cloned into a pGEM-T Easy vector (Promega Corp., Madison, Wisconsin, USA). From each cloning reaction, 4−12 colonies were selected for Sanger sequencing on ABI 3730xl DNA Analyzers (Applied Biosystems, Carlsbad, California, USA). Newly obtained sequences have been deposited to the GenBank (Table 1).

Algal 18S rDNA sequences were edited to remove the plasmid and PCR primer regions and were aligned manually to an alignment used in our earlier study [17] using Mesquite ver. 2.75 [29]. All other green algal sequences were obtained from GenBank. Representative chlorophycean algae were chosen as a diverse sampling of available sequences for this group. Ambiguously aligned sequence regions were removed. The final alignment was used for phylogenetic analyses under likelihood and parsimony criteria. Maximum likelihood (ML) analysis was performed using RAxML ver. 8.0.0 [30], under the GTR+gamma+I model, which was selected using Modeltest ver. 3.7 [31]. ML trees were inferred through 100 iterations, each started from different randomized stepwise addition parsimony trees. The sequences were also analyzed by the maximum parsimony method using PAUP* [32]. Bootstrap analyses were based on 1,000 replicates. Uncorrected (“p”) pairwise distances of amphibian green algal 18S rDNA were calculated using PAUP* [32]. Sequence alignments—both masked and unmasked—used in this study are available as Data S1, S2.

## Results

### 18S rDNA analysis of green algal symbionts

Phylogenetic analyses of 18S rDNA show that all sequences (n = 126) of algae sampled from amphibian egg capsules in this study are clustered together within the Chlamydomonadales, forming a well-supported clade together with *Chlamydomonas pseudogloeogama*, *C. nasuta*, and chlamydomonad sp. strain NDem9/21T-11d (Figure 2). Within this clade, four subclades, which we name I−IV, were identified. Sub-clades I, II, IV correspond to symbiotic green algae of *A. maculatum*, *A. gracile*, and *L. sylvatica*, respectively, whereas clade III includes green algal sequences obtained from *A. maculatum* and the parapatric *L. aurora*. *C. pseudogloeogama* is sister to subclade IV and *C. nasuta* branches with a clade comprising subclades III, IV, and *C. pseudogloeogama*. The chlamydomonad sp. strain NDem9/21T-11d is sister to subclades I+II. Pairwise distance analyses of 18S rDNA indicate that sequences among different subclades differ by 0.65−4.22% (Table 2).

**Table 2:** Percentage pairwise distances (uncorrected) of 18S rDNA among the *Oophila* subclades I−IV.

	II	III	IV
I	0.65−1.72%	3.09−4.16%	3.27−4.22%
II	-	2.97−3.58%	3.15−3.62%
III	-	-	1.37−1.88%

A total of 1,685 nucleotide positions were included for the analysis.

In addition, we obtained 18S rDNA data from three stock algal strains of *Chlamydomonas gloeophila*, two of which, SAG 12−4 and 12−5, were isolated from *A. maculatum* egg masses in 1953 by Starr. The other strain, CCAP 11/127, was isolated from a freshwater pond in Cumbria, England in 1992 by Jaworski. Sequences of the three *C. gloeophila* strains were very similar to each other (Figure 2), but they branched with *C. perpusilla*, and not with or within the ‘*Oophila*’ clade.

### Culturing and observation of green algal symbionts of *A. maculatum*

We have isolated and established strains of algae associated with two geographically distinct *A. maculatum* populations. Note that while the cultures are monotypic in terms of 18S rDNA sequence diversity, they may not necessarily be clonal. The algae grew in AF6 liquid medium [27], although their growth rate was relatively low compared to strains of other chlamydomonad taxa such as *C. pseudogloeogama* (strain SAG 15.73) and *C. nasuta*, (strain NIES-2225). In comparison to these same taxa, the *A. maculatum*-derived algae grew extremely poorly on agar-solidified AF6 media. Despite observing some growth of algae in solid media, the majority of algal colonies did not match the symbiont algal 18S rDNA sequences and instead were matched to other green algae, with 99% or greater sequence similarities, such as *Chlamydomonad sp.* Tow9/21T-1w (AY220568.1), *C. sp*. Tow8/18T-1w (AY220567.1), *C. debaryana* (FR865523.1) and *Chlorococcum minutum* (JN968585.1). This indicates the presence of non-*Oophila* symbionts that outcompeted numerically dominant *Oophila* under solid growth conditions and therefore agar-solidified media should not be used to isolate *A. maculatum*-associated algae.

**Figure 3:**
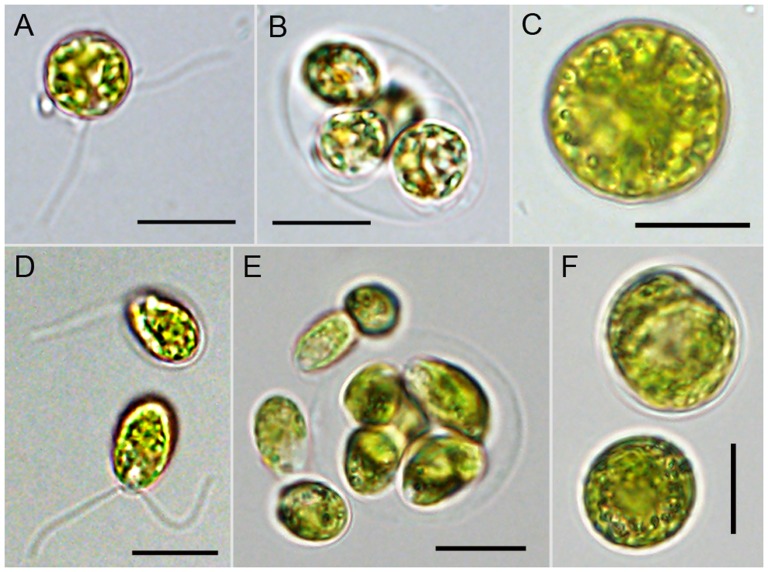
Light microscopic images of cultured strains of *A. maculatum* algae. The *Oophila* strains Hb_cul-rk (A−C) and BB_cul-B (D−F) belong to subclades I and III, respectively. Monotypic cultures displayed at least three different cell types, which include 1) free-swimming biflagellates (A, D), which correspond to zoospores or gametes, 2) cells enclosed within a mother cell wall (B, E), likely representing asexually dividing zoospores, and 3) larger non-motile zygotes (C, F). Scale bars: 10 µm (A−F).

Our cultured strains of *A. maculatum* algal symbionts fall into two distinct subclades, I and III (Figure 2). Algae from both subclades appear to display the canonical chlamydomonad life cycle [33], consisting of vegetative cells (zoospores or gametes, and zygotes (Figure 3). Older cultures tend to have more putative zygotes, the formation of which may be a result of nutrient depletion, as in *C. reinhardtii* [34]. A major difference between strains of distinct phylogenetic groups, which were established and maintained using the same culturing method, is the shape of the zoospores (or gametes). Flagellated cells of the strain belonging to the subclade I are spherical and 9−11 µm in diameter (n = 10), whereas those of the clade II are oblong in shape and measure 10−11 µm in length and 6−7 µm in width (n = 10). Both types of strain are characterized by zoospores having two flagella, which are each nearly twice the length of the cell (Figure 3). Vegetative cell growth of these algae occurs within a parental cell wall, each containing up to eight daughter cells, which are released freely into the media.

## Discussion

We have used sequences derived from DNA amplified using “universal” eukaryotic primers for 18S rDNA to infer the genetic diversity of single celled eukaryotic algal taxa associated with egg masses of four North America amphibian taxa. We acknowledge that a single gene approach can be limited in terms of resolving detailed relationships among closely related samples [35]. However, our 18S rDNA phylogeny reveals that: (i) symbiotic green algae sampled from egg capsules from four amphibians from two different coasts of North America form a distinct, well-supported phylogenetic clade together with three other chlamydomonad taxa within the Chlamydomonadales, (ii) there is a partial relationship between host taxonomic identity and symbiont genotype, and (iii) there are at least two distinct morphological types of algal symbiont found in these amphibian egg masses, and these correspond to phylogenetically distinct lineages. We discuss each of these findings in turn.

### Designation of the ‘*Oophila*’ clade

Our study shows that algal symbiont sequences obtained from broadly distributed populations of North American amphibians are similar to each other and form a well-supported clade together with three chlamydomonad taxa (Figure 2). We designate this group as the ‘*Oophila*’ clade based on the fact that the majority of sequences belonging to the group correspond to symbionts found in amphibian egg masses. Within the ‘*Oophila*’ clade, amphibian algae were further divided into four distinct subclades; some of these subclades display substantial genetic divergence within the 18S rDNA region (e.g.>3% between the subclades I and IV). In the broader phylogenetic context of chlamydomonads, the *Oophila* clade is nested within the Moewusinia clade [36].

Three free-living chlamydomonad taxa are included in the ‘*Oophila*’ clade. These include 1) *Chlamydomonas pseudogloeogama*, which is sister to subclade IV (100% bootstrap support, ML), 2) *C. nasuta*, which branches with subclades III+IV, and 3) the chlamydomonad strain NDem9/21T-11d, which is sister to subclades I+II (Figure 2). The *C. pseudogloeogama* isolate reported in Hoham et al. [37] was SAG 15.73, originally isolated from “snow detritus” in the High Tatra mountains of Slovakia in 1963 by F. Hindák. *C. nasuta*, whose identity has recently been verified by 18S rDNA sequence [38] was isolated from soil samples from Connecticut, USA. Finally, the chlamydomonad sp. NDem9/21T-11d was isolated from a freshwater body located in Itasca State Park of Minnesota, USA by Fawley and colleagues (the sequence has been deposited in GenBank without an associated journal publication). It is currently not clear whether these free-living taxa could form symbioses with amphibian embryos. If so, that would suggest the presence of lineage-specific traits that may allow this algal association. Alternatively, association with amphibian embryos may have arisen independently within the ‘*Oophila*’ clade, or was lost in these free living “species.”

The suggestion that *Oophila* is environmentally acquired as an egg mass symbiont [5], [39], [40], requires that it be a cold tolerant species capable of overwintering in peri- or sub-zero temperatures and then emerging in a planktonic form in the early spring. This is consistent with observed temperature tolerances of *Oophila* in our cultures, which showed growth even under refrigerated conditions with the temperature as low as 6°C. In this sense it is notable that *C. pseudogloeogama*, which is closely related to symbiotic algae of amphibian egg masses (Figure 2), is a cold adapted species. Thus, whereas the original collection site for *C. pseudogloeogama* is geographically distant from those for algae associated with embryos of North American amphibians, their close genetic distance may reflect similarities in their capacity to survive cold climates. The life cycles of neither *O. amblystomatis* nor *C. pseudogloeogama* have been carefully verified, but appear comparable to that of *C. moeuwsii* [33], which is closely related to the ‘*Oophila*’ clade (Figure 2).

### *Oophila* is numerically dominant in *A. maculatum* egg caspules

Environmental sequencing using universal PCR primers for eukaryotic 18S rDNA [28] should amplify DNA sequences from a diverse array of eukaryotes in a sample, thus allowing a relatively unbiased sampling of taxa that would otherwise be difficult to detect and quantify using microscopy. All sequences returned from an environmental sequencing approach fell into the ‘*Oophila*’ clade (Figure 2), which includes an *Oophila* 18S rDNA sequence from our earlier study [17]. Although *Oophila* appears to be the most abundant green alga in *A. maculatum* egg capsules, our sampling strategy was not likely (nor was it meant) to detect rare taxa, and thus we cannot conclude that *Oophila* is the sole algal symbiont. Specifically, we chose a sampling strategy in which we sequenced 18S rDNA from a range of samples from geographically distant locales and distantly related hosts (i.e. frogs vs. salamanders), rather than sequence deeply from fewer samples. For example, if a non-*Oophila* taxon were present in a particular egg capsule at an incidence of 1% (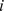 = 0.01) of the population of *O. amblystomatis*, one hundred clones, on average, would need to be sequenced in order to detect this taxon for a given sample. However, we sampled numerous egg capsules from different egg masses, yielding a total of 126 sequences. Assuming equal incidence of non-*Oophila* taxa among all samples and no amplification bias during PCR, the cumulative probability (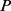) of detecting one non-*Oophila* sequence can be estimated by:
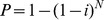
(1)where 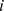 =  the incidence (proportion) of non-*Oophila* taxa in an egg capsule and 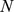 =  the number of sequences. Expressing with respect to 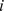:
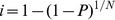
(2)based on our sampling regime, the mean incidence of non-*Oophila* taxa among *A. maculatum* capsules sample would need to be 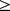 0.068 (6.8%) in order to observe at least one non-*Oophila* sequence with a probability>0.99 (Table 3).

**Table 3:** Maximum incidence of non-*Oophila* taxa in egg capsules, based on a>0.99 cumulative probability of detecting sequences and the actual number of sequences obtained (see equation 2 in Discussion).

Maximum incidence per host				Combined maximum incidence
*A. maculatum*	*A. gracile*	*L. aurora*	*L. sylvatica*	-
0.068	0.156	0.601	0.318	0.036

We thus conclude that *Oophila* is numerically the most abundant algal taxon in *A. maculatum* egg capsules. Although *Oophila* is also the only taxon detected from *A. gracile*, *L. sylvatica* and *L. aurora* egg capsules, our sample size from these taxa does not permit the same strength of conclusion as with *A. maculatum* (Table 3). Using different sampling techniques Kerney et al. [17] reported that the community in egg capsules of *A. maculatum* is not monotypic with respect to green algae. Gilbert [4] and Graham et al. [16] noted spherical and ovoid cells in a single clutch, raising the possibility of either phenotypic plasticity within the population of *Oophila* in the capsule, the co-existence of multiple, distinct green algal taxa, or the presence morphologically distinct subclades of *Oophila* within a single *A. maculatum* egg capsule or egg mass. The latter is supported by our data; in two *A. maculatum* egg capsule samples (GSa & GSb), both the subclade I and III sequence types were identified (Figure 2). In addition, our culturing work, especially the experiment that involved the use of agar solidified media, suggested the presence of non-*Oophila* green algae, albeit numerically not dominant, in *A. maculatum* egg masses. The abundance of other algal taxa relative to *Oophila* as well as the full extent of their diversity, however, remains unknown. Additional sequencing in combination with microscopic cell counting will be necessary to quantify the relative abundance of *Oophila* to other taxa that co-exist in single egg capsules.

Two possibilities can explain the result that the *Oophila* complex is the most common algal taxon among these North American amphibian hosts. One possibility is that algae belonging to the *Oophila* species complex are common and abundant in North American amphibian breeding habitat. DNA sequences that fall into ‘*Oophila*’ clade I have been identified from two *A. maculatum* breeding pools before and during the breeding season (CDB and YL, unpublished data), but the abundance and commonality of free-living *Oophila* in breeding habitats remains to be assessed. A second possibility is that taxa of the *Oophila* clade have evolved into specialist colonizers of amphibian egg masses, such that their distribution patterns coincide with host ranges. Further characterization of host specificity, or identification of *Oophila* from vernal pools that do not contain these amphibian egg masses could help resolve these two possibilities.

### Chlamydomonas gloeophila is not Oophila

In addition to gathering sequence data from amphibian egg masses collected in this study, three culture strains of “*C. gloeophila”* were obtained and processed for 18S rDNA sequencing. Two of these were isolated more than 60 years ago from *A. maculatum* egg clutches collected in New York State (USA) and Connecticut (USA). Whereas sequences from these strains match well to the strain of *C. gloeophila* collected from a freshwater sample in England, none of the *C. gloeophila* strains are closely related to the ‘*Oophila*’ clade (Figure 2), but rather are likely members of the Chlorogonia clade [36]. Interestingly, these cultures were established and maintained on agar-solidified growth media, which our culturing experiments showed are not suitable for growing symbiotic green algae of *A. maculatum*. We, thus, suggest that these *C. gloeophila* strains may not correspond to numerically dominant algal symbionts of *A. maculatum*. Rather, these likely represent low abundance green algae that occurred in *A. maculatum* egg masses, which nevertheless might have outcompeted *Oophila* under non-liquid growth conditions.

### Relationship between host identity and symbiont genotype

18S rDNA sequence evolution is too slow to permit correlations between symbiont genotype and geography within a host species. However, it is interesting that the sequences most divergent to those from *A. maculatum* algae were derived from algae from the wood frog *L. sylvatica*, a host with whom both the geographic range and even breeding habit of *A. maculatum* overlaps. With two exceptions (e.g. some algae from the Greenbrook Sanctuary, NJ, USA and from Beaver Bank, NS, Canada, see Table 1), all sequences from *A. maculatum* form a sister group to those from *A. gracile*, an allopatric congener (Figure 2) [41], [42]. This raises the question as to whether the symbionts of *A. gracile* also enter embryonic tissue and cells, in a manner similar to the symbionts of *A. maculatum*. Finally, it is currently unknown whether *Oophila* belonging to subclade III can invade *A. maculatum* embryonic cells as has been shown for *Oophila* belonging to subclade I [17]. Other algal symbioses have shown both repeated convergent origins (e.g. algal symbionts of the ciliate *Paramecium bursaria* [43], or a single origin with subsequent phylogenetic congruence, indicating co-speciation (e.g. *Chlorella* sp. symbionts of the green hydra [44]. A multiple origins model of North American amphibian-algal symbioses is consistent with finding presumably non-symbiotic chlamydomonad green algae within the *Oophila* clade, and a lack of strict host-symbiont phylogenetic congruence. However further analyses of population-level host-symbiont congruence using additional gene markers [45], environmental sampling from vernal pools, and the diversity cell-cell interactions is needed to further clarify how these fascinating ecological, evolutionary, and developmental associations are established and maintained.

## Supporting Information

Data S1 18S rDNA sequence alignments—masked—used in this study.
(FAS)

Data S2 18S rDNA sequence alignments—unmasked—used in this study.
(FAS)

Figure S1 The original, un-collapsed version of ML tree as shown in Figure 2.
(TIF)
