# Supplementary figures and images for: Phylogenetic Analysis of Algal Symbionts Associated with Four North American Amphibian Egg Masses
Source: PLoS One. 2014 Nov 13;9(11):e108915. doi: 10.1371/journal.pone.0108915 (PMC4230919; doi:10.1371/journal.pone.0108915)

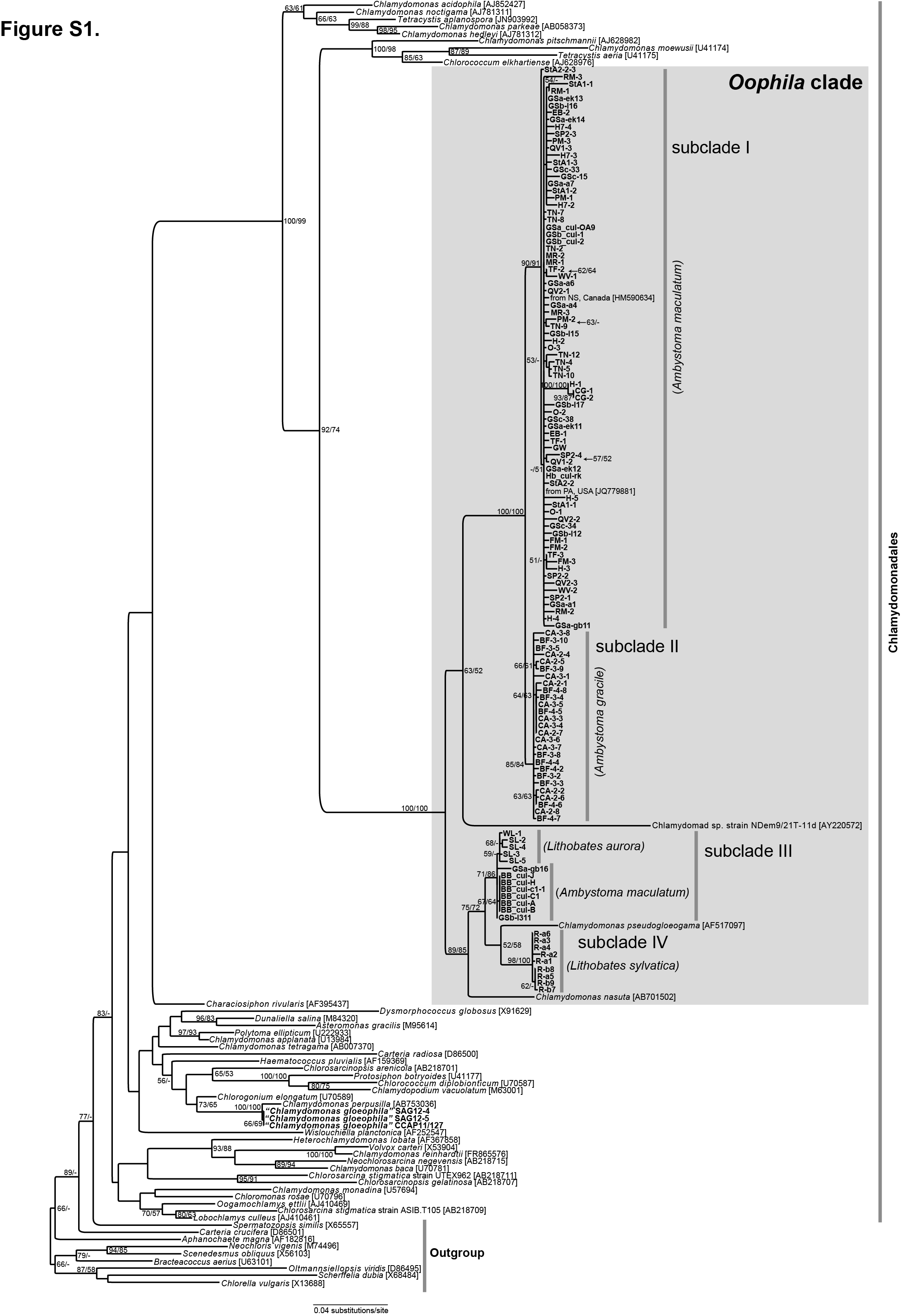

Supplement: Figure S1 — The original, un-collapsed version of ML tree as shown in Figure 2. (TIF) [file pone.0108915.s003.tif]
